# Reversal of New Onset Type 1 Diabetes by Oral *Salmonella*-Based Combination Therapy and Mediated by Regulatory T-Cells in NOD Mice

**DOI:** 10.3389/fimmu.2019.00320

**Published:** 2019-02-26

**Authors:** Jacques C. Mbongue, Jeffrey Rawson, Pablo A. Garcia, Nelson Gonzalez, Jacob Cobb, Fouad Kandeel, Kevin Ferreri, Mohamed I. Husseiny

**Affiliations:** ^1^Department of Translational Research & Cellular Therapeutics, Diabetes & Metabolism Research Institute, Beckman Research Institute of City of Hope, Duarte, CA, United States; ^2^Faculty of Pharmacy, Zagazig University, Zagazig, Egypt

**Keywords:** *Salmonella*, Treg-cells, Tr1-cells, immunotherapy, type 1 diabetes, immunomodulators, oral vaccine

## Abstract

Autoimmune diseases such as type 1 diabetes (T1D) involve the loss of regulatory mechanisms resulting in increased tissue-specific cytotoxicity. The result is destruction of pancreatic insulin-producing β-cells and loss of glucose homeostasis. We are developing a novel oral vaccine using live attenuated *Salmonella* to deliver TGFβ, IL10, and the diabetic autoantigen preproinsulin combined with low-doses of anti-CD3 mAb. Here we show that oral administration of *Salmonella*-based anti-CD3 mAb combined therapy reverses new-onset T1D in non-obese diabetic (NOD) mice. The therapeutic effect of the combined therapy was associated with induction of immune suppressive CD4^+^CD25^+^Foxp3^+^ Treg and CD4^+^CD49b^+^LAG3^+^ Tr1 cells. In adoptive transfer experiments, adding or depleting Treg or Tr1 cells indicated that both are important for preventing diabetes in combined therapy-treated mice, but that Tr1 cells may have a more central role. Furthermore, induced Tr1 cells were found to be antigen-specific responding to peptide stimulation by secreting tolerance inducing IL10. These preclinical data demonstrate a role for Treg and Tr1 cells in combined therapy-mediated induction of tolerance in NOD mice. These results also demonstrate the potential of oral *Salmonella*-based combined therapy in the treatment of early T1D.

## Introduction

Type 1 diabetes (T1D) is a tissue-specific autoimmune disease resulting from lymphocyte destruction of pancreatic islet insulin-producing β-cells (1–3). The loss of islet β-cell function leads to insulin deficiency and high blood glucose levels (hyperglycemia) (4). Defective antigen-presenting cells (APCs), particularly dendritic cells (DCs), in the gut-associated lymphoid tissue (GALT) halt the differentiation of gut associated regulatory T-cells (Tregs) and contribute to T1D-associated immune dysfunction (5). Multiple approaches have and are being pursued to correct or limit this process including monoclonal antibodies, autoantigen immunization, Treg-based immunotherapy and systemic immunosuppressive drugs targeting T cells or antigen presenting cells. However, most of these interventions have been complicated by severe immune suppression or have shown a lack of efficacy (6–8).

The induction and formation of peripheral immune tolerance is crucial to maintaining stability of the immune system. In addition to the immune system's major roles in regulating clonal deletion, antigen sequestration, and expression at privileged sites, a variety of regulatory immune cells have critical roles in maintaining peripheral immune tolerance. Tolerogenic dendritic cells (Tol DCs) are a group of dendritic cells with immuno-suppressive properties, directing the immune system into a tolerogenic state against foreign and self-antigens. These tolerogenic effects are mostly mediated through induction of Tregs, Th3 regulatory cells, Treg 17, and type 1 regulatory T cells (Tr1) (9–14) or through promoting cell anergy or apoptosis. Tr1 cells are capable of suppressing T cell responses and modulating APC function via a variety of mechanisms, including expression of inhibitory cell surface receptors, cytolytic activity, and secretion of soluble factors (15). Over the past two decades, the peripherally derived Treg cell subset CD4^+^Foxp3^−^ (Tr1) cells have received increasing attention, particularly in relation to peripheral immune tolerance. Tr1 cells are induced by chronic activation of CD4^+^ T cells by antigen in the presence of interleukin-10 (IL10) and are thought to represent a new subset of CD4^+^ T cells in humans and mice with the essential markers CD4^+^ CD49b^+^ LAG-3^+^ (16, 17). LAG-3 is a membrane protein on Tr1 cells that negatively regulates TCR-mediated signal transduction (17, 18). LAG-3 has been shown to activate DCs and to promote Tr1 cell antigen specificity (19–21).

Evidence suggests that islet-specific Tr1 cells may play an important role in the regulation of T1D. First identified as a population of naturally arising CD4^+^ T cells that secrete IL10 following exposure to islet autoantigens (22). Tr1 cells are now recognized as playing an important role the pathogenesis of T1D. Intestinal IL10-producing Tr1 cells decrease diabetes incidence in NOD mice (23, 24). In response to activation of several chemokine receptors, including CCR4, CCR5, and CCR7, Tr1 cells suppress proliferation of pro-inflammatory Th1 cells in the pancreas. Additionally, in preclinical models of islet transplantation, Tr1 cell has been shown to induce antigen (Ag)-specific tolerance (25).

Non-pathogenic *Salmonella typhimurium* allows safe and effective delivery of oral antigen-specific vaccines (26, 27). Infection with attenuated S*. typhimurium* has been shown to generate beneficial non-specific immune responses in NOD mice (28). When given orally, *Salmonella* carrying an antigen-expression plasmid are transferred from the gut to phagosomes of GALT antigen presenting cells (APCs) where they form *Salmonella*-containing vacuoles (SCV). The bacteria remain viable and multiply inside the SCV and deliver the recombinant antigen into the host cell cytosol, thus avoiding intestinal degradation of antigen (29–32). The APCs process and present the antigen to other immune cells in the gut then migrate to other organs (33, 34). Such vaccines have been shown to be very effective in eliciting both CD8 and CD4 T cell-mediated immune responses in models of infectious diseases and cancer (35, 36). In fact, *Salmonella* is being used for the development of cancer vaccines with promising results, although maximum effectiveness requires addition of immunostimulatory agents to augment the cytotoxic effect (29).

We previously developed an oral *Salmonella*-based delivery system (SPI2-TTSS) that was found to be effective in delivering autoantigen (preproinsulin, PPI) in combination with TGFβ to prevent diabetes in NOD mice (37). Our studies also showed that the co-administration of a short-course of anti-CD3 mAb along with a reduced dose of *Salmonella*-based vaccine (PPI+TGFβ+IL10) preserved insulin-positive cells, reduced insulitis and prevented autoimmune diabetes in NOD mice (38). Herein, we tested the effect of *Salmonella*-based combined therapy and its mechanism mediated by regulatory cell types involved in vaccine-driven prevention and reversal of new onset diabetes in NOD mice.

## Materials and Methods

### Preparation of *Salmonella* Vaccines

Expression plasmids for autoantigens (mouse preproinsulin (PPI) and immunomodulators (TGFβ and IL10) were prepared as described (37, 38). We also used non-diabetogenic antigen such as listeriolysin O (LlO) from *Listeria* (32) as combined therapy with TGFβ+IL10 and anti-CD3 to orally vaccinate diabetic mice. Bacteria were cultured and allowed to grow to log phase in Luria-Bertani (LB), followed by adjusting its OD_600_ then resuspended in 5% sodium bicarbonate to provide the appropriate dose in a total volume of 200 μL. Bacteria selection was performed by using ampicillin (100 μg/ml), kanamycin and/or carbenicillin (50 μg/ml).

### Animal Experiments

Seven week old female NOD/ShiLtJ (NOD) and NOD.*Cg-Prkdc*^*scid*^ *Il2rg*^*tm*1*Wjl*^/SzJ (NSG) mice obtained from The Jackson Laboratory (Bar Harbor, ME) were maintained under specific pathogen-free conditions. Animals received high quality care consistent with Public Health Service Policy. The animal care facility at City of Hope is fully accredited by the Association for Assessment and Accreditation of Laboratory Animal Care International (AAALAC). The study was approved by the Institutional Animal Care and Use Committee (IACUC# 11032 and 18017). NOD mice (8 weeks old) were vaccinated by oral gavage on days 0 and 7. Furthermore certain cohorts were treated for 5 consecutive days (days 0–4; 2.5 μg i.p. /mouse) with hamster anti-CD3 mAb (clone 145-2C11, Bio-X-Cell, West Lebanon, NH) either alone or in combination with oral vaccine (38). Blood glucose was measured every 3–4 days with One Touch Ultra glucometer (LifeScan, Milpitas, CA). Mice were considered diabetic when blood glucose values exceeded 200 mg/dL for two consecutive measurements.

### Flow Cytometry

Single cell suspensions of spleen were prepared by gentle disruption and tissue digestion in fresh collagenase D (1 mg/ml) at days 21, 28, 49 or 105 post-vaccine treatments depending on the experiment. Cells were stained with conjugated antibodies against CD4 (GK1.5), CD8a (53-6.7) or matching isotype controls (Biolegend), and CD25 (PC61.5) (eBioscience), PerCP/Cy5.5 anti-mouse CD49b (Biolegend), APC anti-mouse LAG3 (Biolegend). Cells were analyzed with a BD Fortessa flow cytometer (BD Biosciences) and FlowJo software 10.4.

### Treg Suppression Assay

Splenocytes were prepared via standard methods. CD4^+^CD25^−^ as responder T-cells (Tresp) were isolated by FACS cell sorting then labeled with 1:1,000 of 5 mM Cell trace Violet (CTV). Suppression assays were performed in round-bottom 96-well plates containing 1 × 10^5^ Tresp cells cultured with or without CD4^+^CD25^+^ Treg cells, isolated from either vehicle or vaccine-treated groups by FACS cell sorting, using decreasing cell ratios including (0:1, 1:1, 1:2, 1:4, and 1:8). Then the cells cultured with anti-CD3/anti-CD28-coated beads at a ratio of 1:1 according to Mouse Dynabead T-activator CD3/CD28 protocol (Gibco life technologies). After 3 days, the proliferation of CD4 responder T cells positive for CTV was analyzed by the conventional method of gating on the proliferated cells using the FlowJo software 10.4.

### ELISA and Cytokine Measurement

CD4^+^CD49b^+^LAG3^+^ Tr1 cells from either vehicle or vaccine-treated NOD mice were cultured with CD3/CD28 latex beads and either 50 μg/ml of insulin peptide B9-23(SHLVEALYLVCGERG, CAT# AS-61532, Anaspec), 50μg/ml of GAD65 (SRLSKVAPVIKARMMEYGTT, p524-543, CAT#AS-62757, Anaspec), or 50 μg/ml of OVA peptide (ISQAVHAAHAEINEAGR, CAT# 323-339, GenScript), and supernatants were collected after 72 h. Mouse IL10 levels in the supernatants and serum circulating levels of IL10 and TGFβ were quantified using the Mouse Quantikine ELISA Kit (R&D Systems) according to the manufacturer's instructions.

To assess antigen-specificity, splenocytes (5 × 10^5^ cells) obtained from different treated groups of mice followed by *in vitro* stimulation by culturing with insulin peptide B9-23 for 72 h. The levels of IFNγ, TNFα, IL12p70, and IL17A were quantified in cell-free supernatants using a ProcartaPlex kit (eBioscience) and Bio-Plex analyzer (Bio-Rad, Hercules, CA).

### Adoptive Transfer of Diabetes

In experiments using unfractionated splenocytes, 1 × 10^6^ pooled splenocytes from diabetic, vehicle or vaccine-treated NOD mice were transferred into NSG recipient mice. Fractionated cells were used in certain cases including CD4^+^CD25^+^ T-cells isolated from spleens of vehicle or vaccine-treated NOD mice using CD4^+^CD25^+^ Regulatory T Cell Isolation Kit (Miltenyi Biotec), or Tr1 cells isolated by FACS through sorting of CD4^+^CD49b^+^LAG3^+^ cells. The regulatory cells and the depleted cell fractions were collected separately. 1 × 10^5^ regulatory cells of either type were combined with 1 × 10^6^ splenocytes from overtly diabetic NOD mice and transferred into NSG recipient mice. In depletion experiments, 3 × 10^6^ splenocytes from either vehicle or vaccine-treated mice which were depleted from Treg or Tr1 cells and transferred into NSG recipient mice. Blood glucose levels were monitored as described before.

### Statistical Analyses

Survival analyses with Kaplan-Meier estimates were used to evaluate the incidence of diabetes between groups with differences determined by Mantel-Cox log-rank test analysis. One-way or two-way ANOVA were used for analysis of percentage of positive cells between groups and to compare cell populations after FACS analysis. A *p* < 0.05 was considered significant. Statistical analysis was performed using GraphPad Prism 7 software.

## Results

### *Salmonella*-Based Combination Therapy Reverses Autoimmune Diabetes in NOD Mice

Our previous study showed that 2 doses of *Salmonella*-based combined therapy was essential for prevention of diabetes in NOD mice (38).

**Figure 1:**
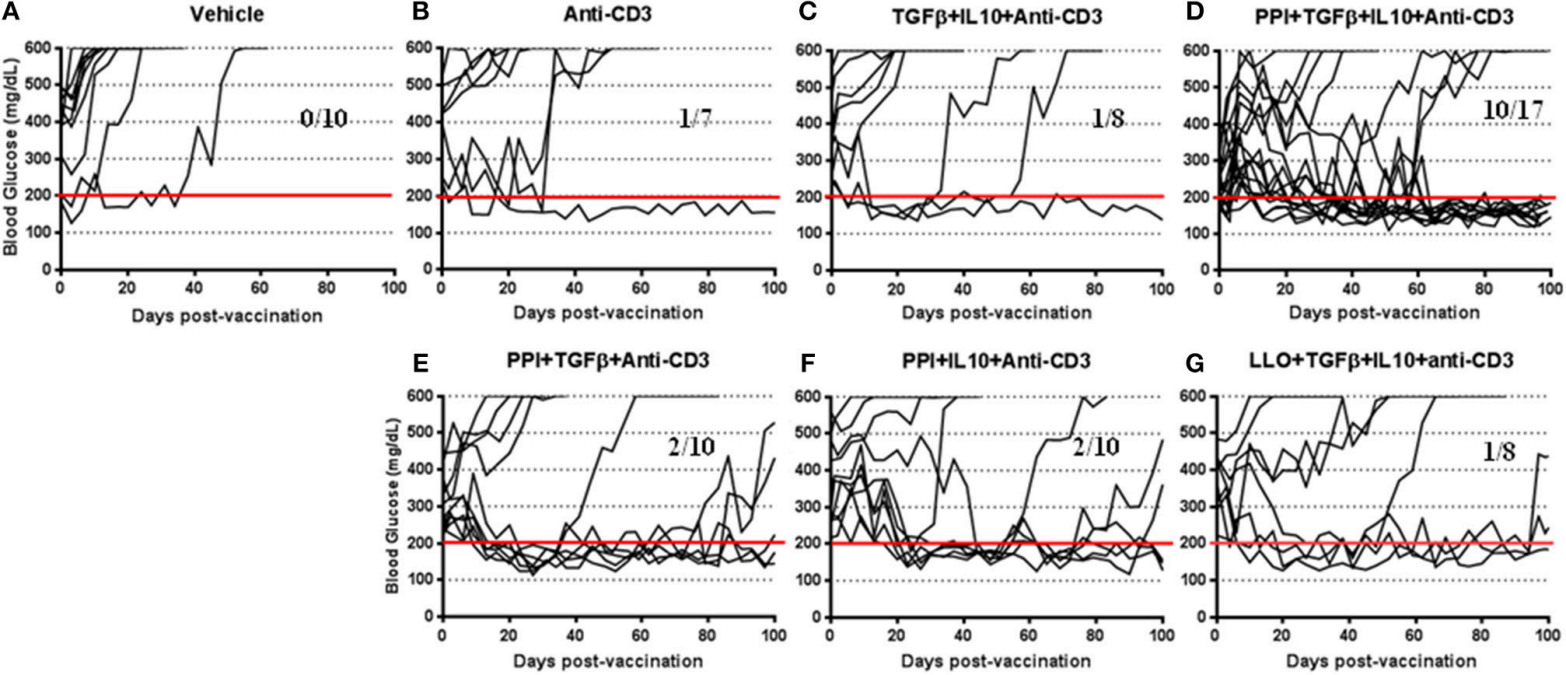
*Salmonella*-based combination therapy reverses diabetes in NOD mice. Female new onset diabetic NOD mice were treated via intraperitoneal (i.p.) injection with five once-a-day doses of anti-CD3 mAb (2.5 μg/mouse). Then mice were orally vaccinated with vehicle **(A)**, anti-CD3 alone **(B)**, TGFβ+IL10+anti-CD3 **(C)**, PPI+TGFβ+IL10+anti-CD3 **(D)**, PPI+TGFβ+anti-CD3 **(E)**, PPI+IL10+anti-CD3 **(F)**, and non-diabetogenic LLO+TGFβ+IL10+anti-CD3 **(G)** for 2 consecutive weeks starting one day after conclusion of anti-CD3 therapy. Data displayed as the blood glucose levels in NOD mice for 100 days after vaccine start.

To further investigate the therapeutic effect of the *Salmonella*-based vaccine, early diabetic NOD mice (when two consecutive blood glucose level over 200 mg/dL) were vaccinated by oral gavage on days 0 and 7. Additionally, certain cohorts were treated for 5 consecutive days (days 0–4; 2.5 μg i.p. /mouse) with anti-CD3 mAb. Figure 1 showed that all 10 diabetic mice of the vehicle treated group were hyperglycemic (Figure 1A), only one diabetic mouse out of 7 from the anti-CD3 treated group was reversed (Figure 1B), and only one mouse out of 8 from the group treated with TGFβ+IL10+anti-CD3 was reversed (Figure 1C). Conversely, 10 out of 17 diabetic mice treated with combined vaccine therapy were reversed and become euglycemic and stable for about 100 days post-vaccination (Figure 1D). Furthermore, combination therapy without IL10 was effective in reversing 6 out of 10 diabetic mice by 2 weeks post-vaccination (Figure 1E), however reversion to a diabetic state occurred in 3 animals after 100 days post-vaccination. In addition combination therapy without TFGβ was effective in reversing diabetes in 2 mice out of 10 mice by 100 days post-vaccination (Figure 1F). On the other hand 7 out of 8 diabetic mice from the group treated with combination therapy using non-diabetogenic antigen listeriolysin O (Llo) were still diabetic (Figure 1G).

*Salmonella*-based combination therapy was significantly increased the tolerogenic regulatory cytokines such as IL10 and TGFβ in the serum of vaccinated mice (Supplementary Figure 1). Interestingly, levels of the pathogenic inflammatory cytokines IFNγ, TNFα, IL12p70, and IL17A were inhibited by combination therapy containing IL10 after *in vitro* stimulation of splenocytes with Insulin peptide B9-23 (Supplementary Figure 2). Finally, vaccination in combination with PPI+TGFβ+IL10 and anti-CD3 mAb was found to be most effective and specific in reversing new onset diabetes (Figure 1).

### *Salmonella*-Based Combination Therapy Induces Suppressive Tregs

**Figure 2:**
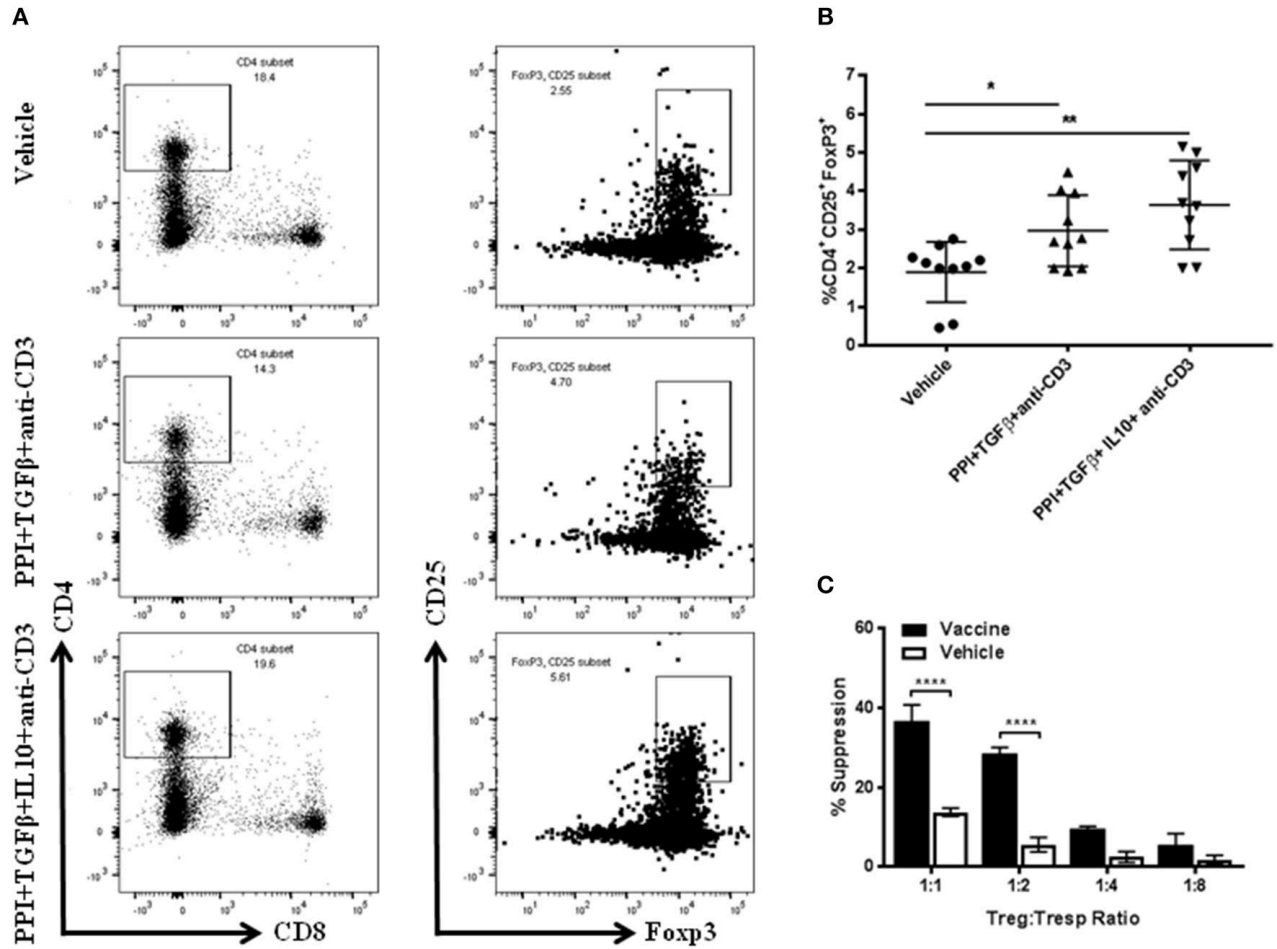
*Salmonella*-based combination therapy induces suppressive Tregs. Female 7–8 week old NOD mice were treated with 2.5 μg/mouse of anti-CD3 mAb via i.p. injection for 5 days followed by 2 doses of oral combined vaccines. Splenocytes were harvested from the indicated group at day 30 post-vaccination. **(A)** Representative FACS plots gated on live CD4 T cells indicate the frequency of CD4^+^CD25^+^Foxp3^+^ T cells in the spleens of mice from each group. **(B)** Quantification the percentages of CD4^+^CD25^+^FoxP3^+^ Tregs in each treated group. Data is presented as means ± *SD* are from 2 independent experiments. Statistical analysis using one-way ANOVA shows the significance between combined therapy and vehicle group (*p < 0.05; **p < 0.01). **(C)** *in vitro* suppression assay of Treg in culture with CD4^+^CD25^−^ T responder cells and CD3/CD28 beads. Statistical analysis using two-way ANOVA shows the significance between combined therapy and vehicle group (****p < 0.0001).

To identify the specific cell types that were involved in the vaccine-mediated prevention of diabetes, FACS analysis of splenocytes CD4^+^CD25^+^Foxp3^+^ Tregs from NOD mice treated with the combination therapy with or without IL10 was performed (Figure 2A). Greater percentages of regulatory CD4^+^CD25^+^Foxp3^+^ cells were present in the spleens of mice treated with combination therapy compared to those treated with vehicle (one-way ANOVA, *p* = 0.008, Figure 2B). Regulatory CD4^+^CD25^+^Foxp3^+^ cells in mice treated with combination therapy without IL10 were also increased compared with those treated with vehicle (one-way ANOVA, *p* = 0.01). The highest level of Tregs was observed in mice treated with the combination therapy indicating a correlation between Treg induction and vaccine diabetes prevention and reversal (Figure 2). Furthermore, the functional capacity of the Tregs isolated from animal treated with combined immunotherapy was assessed. The results showed that the CD4^+^CD25^+^ T cells from vaccine-treated mice effectively suppressed the proliferation of polyclonally stimulated CD4^+^CD25^−^ Tresps in an *in vitro* suppression assay (Figure 2C).

**Figure 3:**
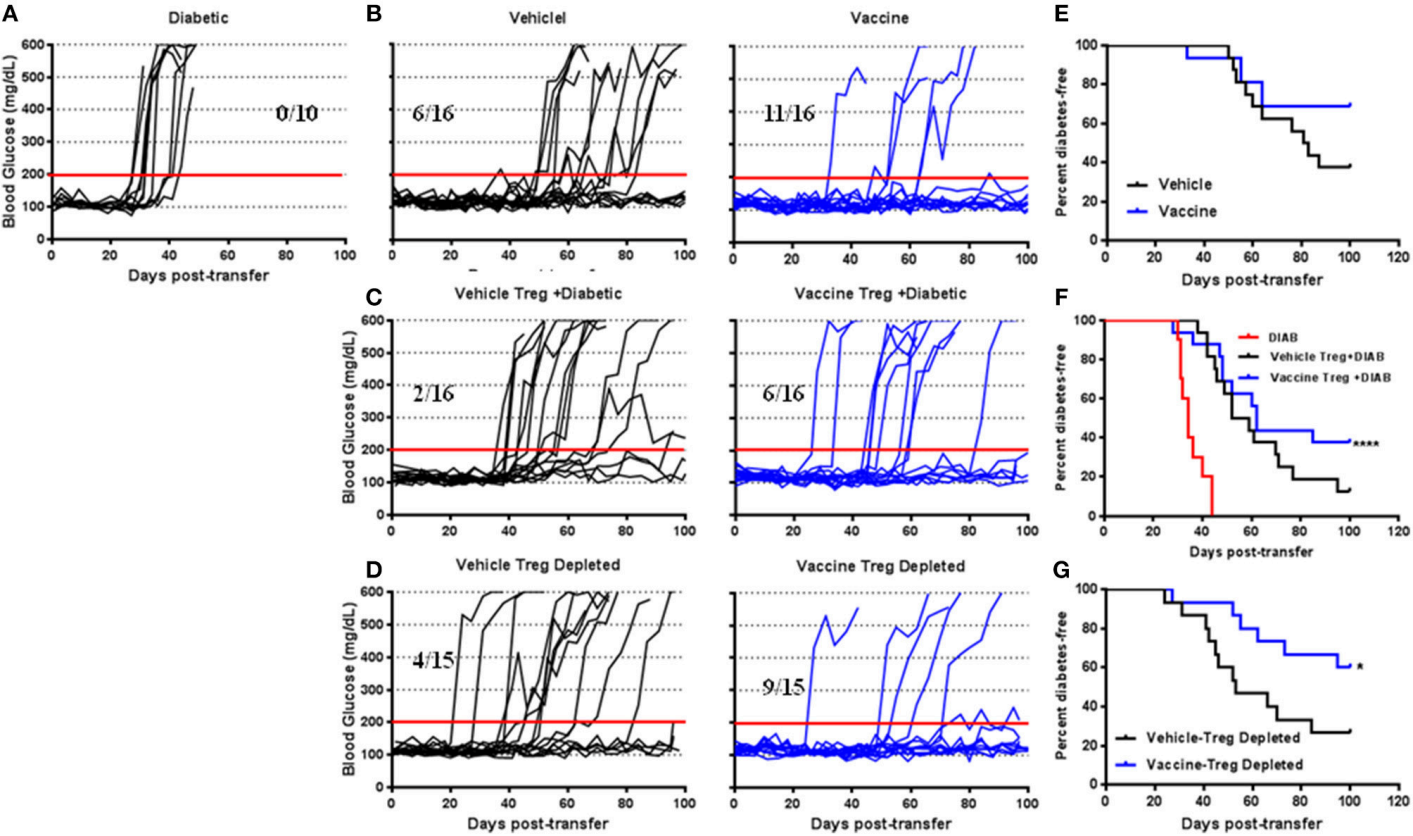
The role of *Salmonella*-based combination therapy induced Tregs in suppression of autoimmune diabetes. Female 7–8 week old NOD mice were treated with 2.5 μg/mouse of anti-CD3 mAb via i.p. injection for 5 days followed by 2 doses of oral combined vaccine. Splenocyte pools were prepared from the indicated treated groups at day 30 post-vaccination. **(A)** Adoptive transfer of splenocytes from overtly diabetic NOD mice into recipient NSG mice. **(B**,**E)** Adoptive transfer of splenocyte pools from NOD mice treated with vehicle or combined therapy into NSG mice. **(C**,**F)** Co-transfer of splenocyte Tregs from vehicle or vaccinated mice with diabetic splenocytes into NSG recipient mice. **(D**,**G)** Adoptive transfer of Treg-depleted splenocytes isolated from vehicle or vaccinated NOD mice into NSG mice. **(A**–**D)** Data display the blood glucose levels for individual mice over the time. **(E**–**G)** Data presented as the Log-rank plot of the percentage of NSG mice that remained diabetes-free over the time after transfer. The differences between the group of mice vaccinated with combined therapy and vehicle was significant (*p < 0.05, ****p < 0.0001) by the log-rank (Mantel-Cox) test.

To define the suppressive activity of CD4^+^CD25^+^Foxp3^+^ Tregs in the vaccine-mediated effects adoptive transfer experiments were performed (*in vivo* suppression assay). NSG mice injected with splenocytes isolated from diabetic NOD mice were developed diabetes in all cases within 40 days post-transfer (Figure 3A). on the other hand, NSG mice that received splenocytes from NOD mice 4 weeks post-vehicle treatment were developed diabetes in 10 out of 16 cases (Figure 3B). Conversely, animals receiving splenocytes from vaccinated NOD mice developed diabetes in 5 out of 16 cases (Figure 3B). Furthermore, the splenocytes from vaccinated mice decreased the incidence of diabetes in NSG mice more than the splenocytes from vehicle-treated mice (Figure 3E). Co-transfer of CD4^+^CD25^+^ Tregs isolated from spleens of vehicle-treated NOD mice with diabetic splenocytes resulted in a higher incidence of diabetes in recipient mice (14 out of 16) than that found in a animals given cells from vaccine-treated mice (10 out of 16, Figure 3C). This suggests that with Tregs from vaccinated mice were effective at limiting diabetes compared with Tregs from vehicle-treated mice (Figure 3F) (Log-rank (Mantel-Cox) test, *p* < 0.0001). However, transfer of Treg-depleted splenocytes isolated from vehicle-treated mice resulted in the loss of this protection against diabetes in recipient mice (11 out of 15, Figure 3D). When the same was done with splenocytes from vaccinated animals, 9 out of 15 the NSG recipient mice remained euglycemic for over 100 days (Figure 3D). Moreover, the splenocyte-depleted Tregs was significantly decreased the incidence of diabetes in recipient mice compared to mice receiving cells from vehicle-treated animals (Log-rank (Mantel-Cox) test, *p* = 0.04) (Figure 3G).

### *Salmonella*-Based Combination Therapy Induces Tr1 Cells

**Figure 4:**
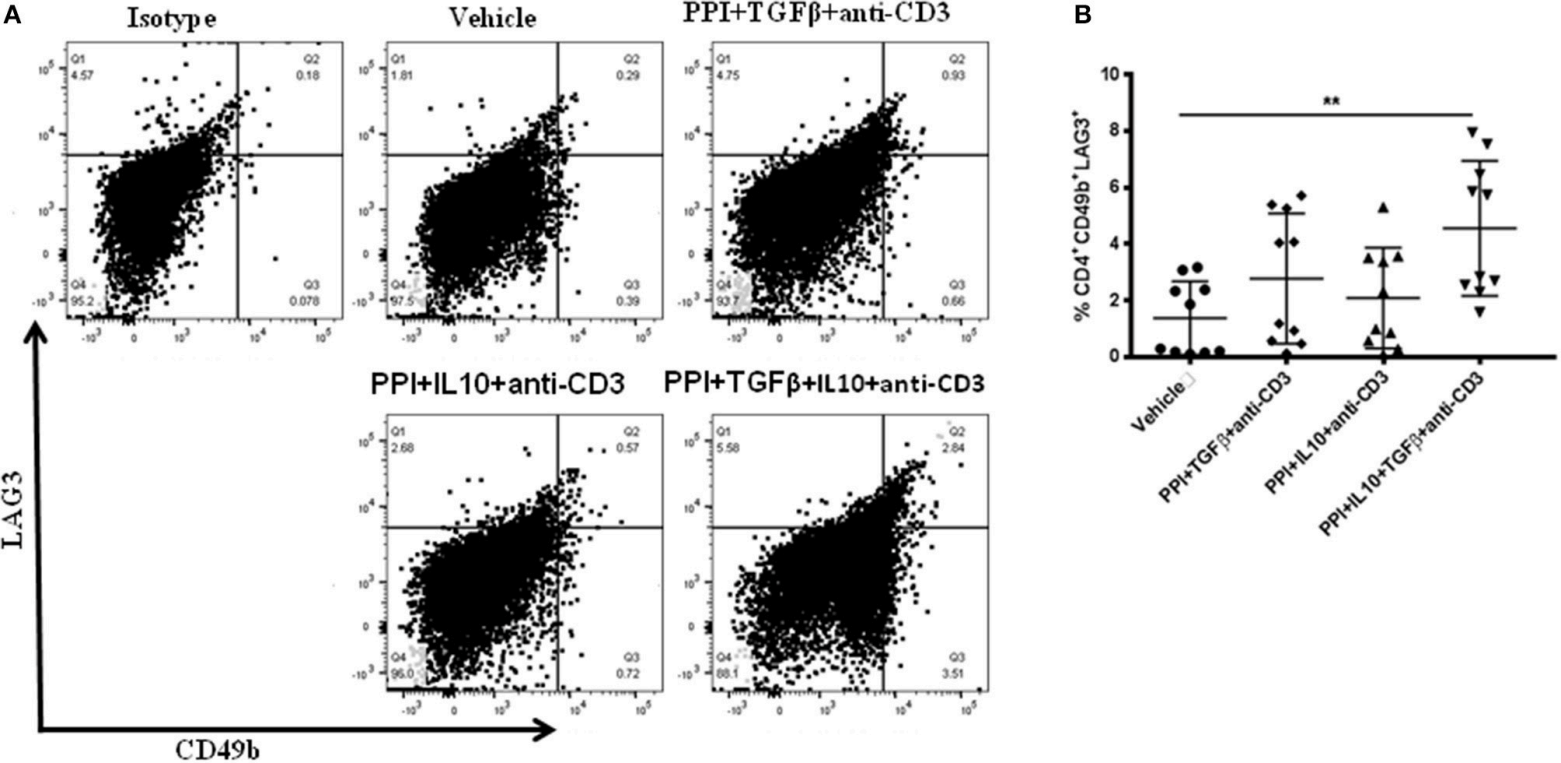
*Salmonella*-based combination therapy induces Tr1 cells in NOD mice. Female 7–8 weeks old NOD mice were treated orally with 2 doses of combined vaccine and with 2.5 μg/mouse of anti-CD3 mAb as described. Splenocytes were prepared from the indicated treated groups at day 30 post-vaccination. **(A)** Representative FACS plots gated on live CD4 T cells indicate the frequency of CD4^+^CD49b^+^LAG3^+^ T cells in the spleens of mice from each treatment group. **(B)** Quantification the percentages of CD4^+^CD49b^+^LAG3^+^ T cells in each treated group (10 mice each). Data presented as means ± *SD* from 2 independent experiments. Statistical analysis using one-way ANOVA shows the significance between combined therapy and control group (**p < 0.01).

Treg-depleted splenocytes from vaccinated mice was lowered the incidence of diabetes in recipient mice (Figures 3D,G) and suggests a role for other regulatory cell types in this effect. CD4^+^CD49b^+^LAG3^+^ type 1 regulatory (Tr1) cells are important for controlling autoimmunity and could be involved. Tr1 cells levels were measured in splenocytes from NOD mice treated with the combination therapy with or without TGFβ or IL10 using flow cytometry and gated on live CD4^+^ T-cells (Figure 4A). CD4^+^CD49b^+^LAG3^+^ Tr1 cells were induced within 2 weeks after vaccination with the highest levels of Tr1 cells were obtained from animals treated with the combination therapy lacking IL10 or TGFβ (one-way ANOVA, *p* = 0.003) (Figure 4B). Interestingly Tr1 cells were measured via flow cytometry in splenocytes from NSG mice after adoptive transfer (Supplementary Figure 3A). CD4^+^CD49b^+^LAG3^+^ Tr1 cells were found significantly increased in spleens of recipient mice that had received either or Treg-depleted splenocytes from vaccine-treated mice compared to mice given cells from vehicle-treated animals (Supplementary Figure 3, one-way ANOVA, *p* = 0.0001, *p* = 0.0002). Collectively, induction of Tr1 cells occurs concomitantly with Tregs and is intimately dependent on the combination of TGFβ and IL10.

### Long-Term Induction of Tr1 in NOD Mice by the Combination Therapy Requires IL10

**Figure 5:**
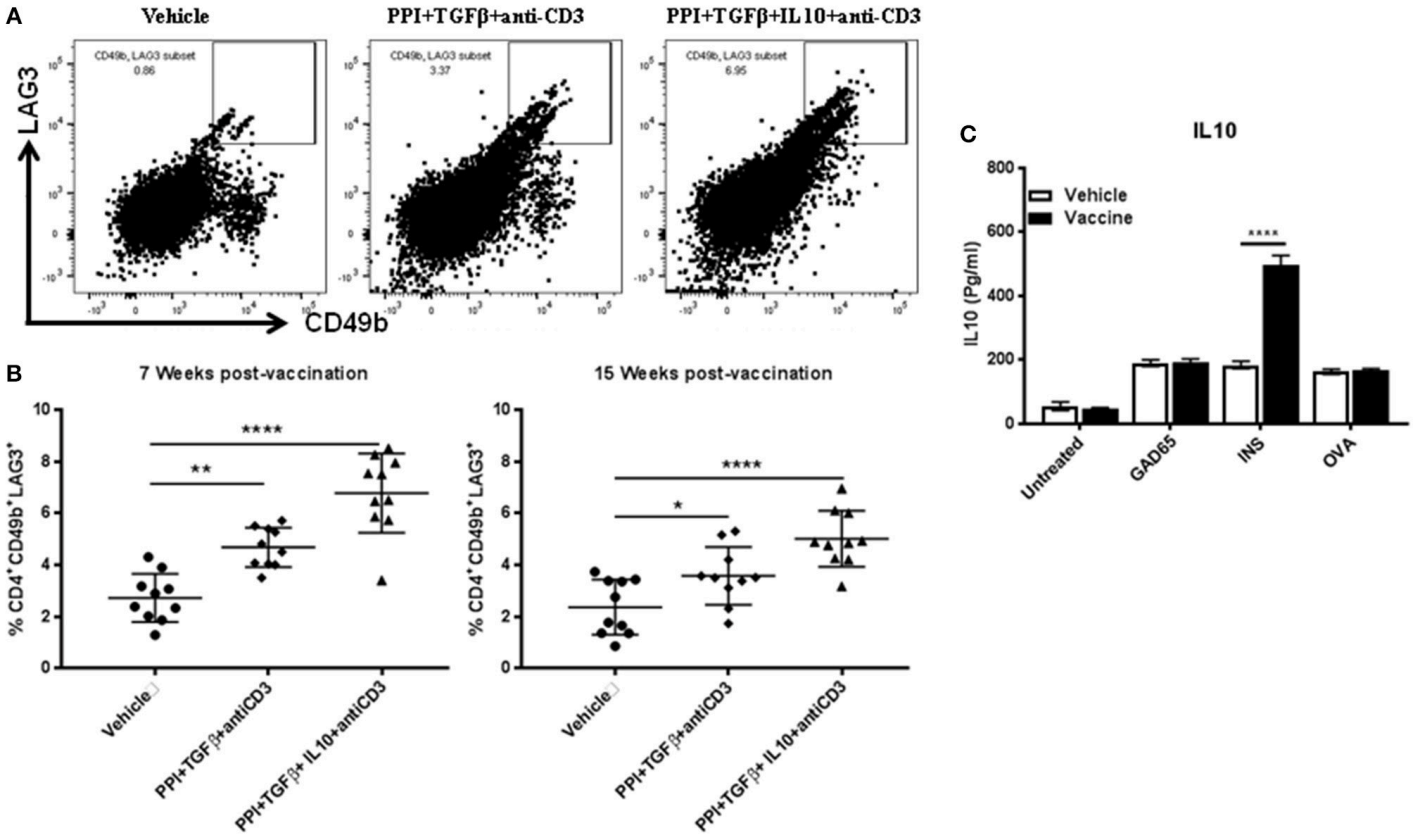
IL10 is required for long-term Tr1 induction in NOD mice following *Salmonella*-based combination therapy. Female 7–8 weeks old NOD mice were treated orally with 2 doses of combined vaccine with 2.5 μg/mouse of anti-CD3 mAb as described. Splenocytes were prepared from the indicated treated groups at 7 weeks and 15 weeks post-vaccination. **(A)** Representative FACS plots gated on live CD4 T cells indicate the frequency of CD4^+^CD49b^+^LAG3^+^ T cells in the spleens of mice from the indicated groups. **(B)** Scatter plots of the percentages of CD4^+^CD49b^+^LAG3^+^ Tr1 expression across the 3 groups at 7 weeks and 15 weeks post-vaccination. Data presented as means ± *SD* from 2 independent experiments. Statistical analysis using one-way ANOVA shows the significance between combined therapy and control group (*p < 0.05: **p < 0.01; ****p < 0.0001). **(C)** *in vitro* stimulation assays of vaccine induced Tr1. Pooled splenocytes CD4^+^CD49b^+^LAG3^+^ Tr1 cells were isolated from vaccinated and vehicle-treated mice 3 weeks post-vaccination by FACS sorting. Secretion of IL10 was determined by ELISA of culture supernatants obtained from CD4^+^CD49b^+^LAG3^+^ Tr1 cells after peptide stimulation for 3 days. Data presented as means ± *SD* and obtained from 4 replicas. The statistical significance was calculated with two-way ANOVA and the significance level indicated by asterisks (****p < 0.0001).

IL10 is required for Tr1 induction (11, 15, 23). Our previous studies have shown that IL10 delays the onset of diabetes in NOD mice treated with *Salmonella*-based vaccine (38). we now find that IL10 is essential for stabilization of blood glucose in NOD mice after reversal of diabetes (Figure 1E). To study the role of IL10 in vaccine-mediated Tr1 induction, the levels of Tr1 cells in NOD mice were quantified at 7 and 15 weeks post-vaccination using flow cytometry gated on live CD4^+^ cells (Figures 5A,B). Vaccination was resulted in a significant increase of CD4^+^CD49b^+^LAG3^+^ Tr1 cell levels in spleens of NOD mice by 7 weeks with the highest levels were found in animals receiving IL10 (one-way ANOVA, *p* = 0.002 and *p* < 0.0001, Figure 5B) an advantage that persisted at least through 15 weeks post-vaccination (one-way ANOVA, *p* = 0.04 and *p* < 0.0001).

While vaccination with the combination therapy increases CD4^+^CD49b^+^LAG3^+^ Tr1 cells, it was not clear if these cells carried specificity to a diabetes relevant antigen. To test this, splenocyte CD4^+^CD49b^+^LAG3^+^ Tr1 cells, were isolated from vaccinated and vehicle-treated mice 3 weeks post-vaccination. Following challenged *in vitro* with insulin-derived peptide B9-23. IL10 levels in conditional medium from acquired from vaccinated mice were higher compared to Tr1 cells from vehicle treated mice (two-way ANOVA, *p* < 0.0001). Conversely challenge with GAD65 peptide and OVA peptide had no effect upon cytokine levels in conditional medium (Figure 5C).

### Tr1 Cells Are Essential for *Salmonella*-Based Combination Therapy in Prevention of Diabetes

**Figure 6:**
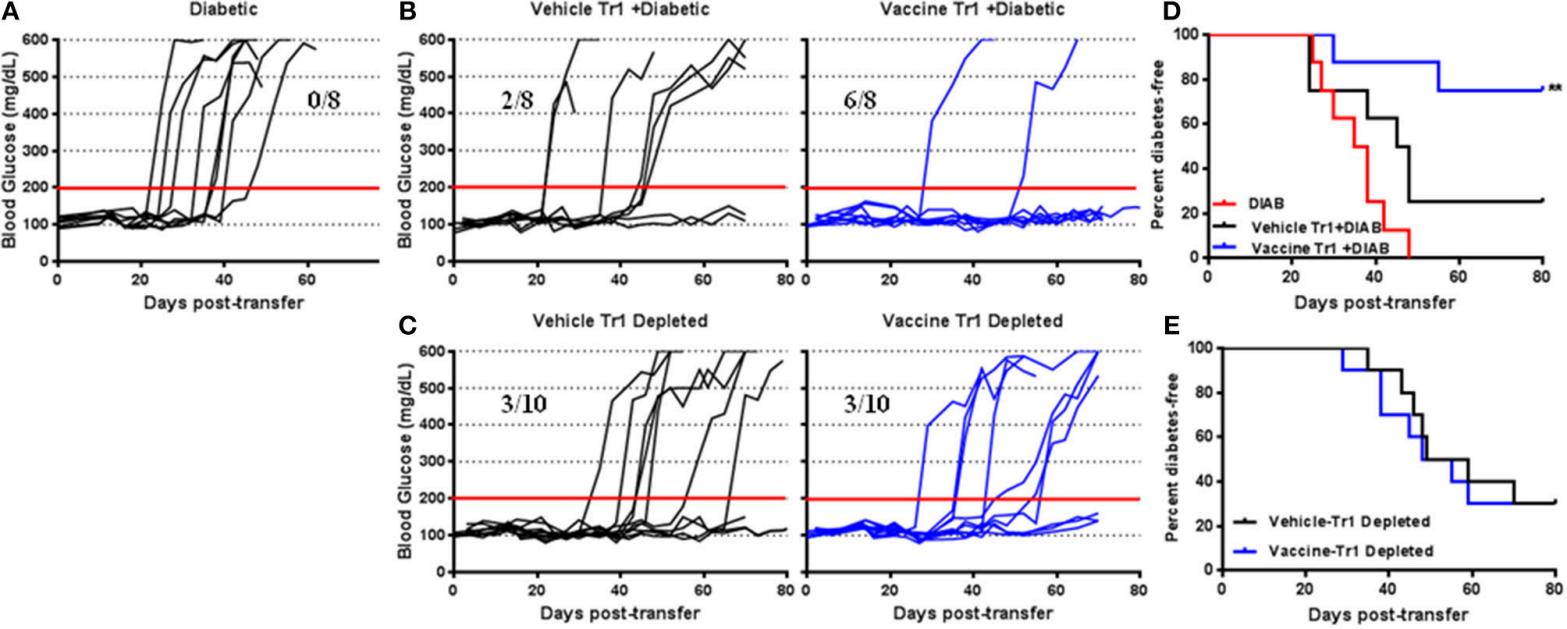
Tr1 cells are crucial for *Salmonella*-based combined therapy of diabetes in NOD mice. Female 7–8 weeks old NOD mice were orally vaccinated as described. Splenocytes were prepared from the indicated treated groups at day 30 post-vaccination. **(A)** Adoptive transfer of splenocytes from overtly diabetic NOD mice into recipient NSG mice. **(B**,**D)** Co-transfer of splenocyte Tr1cells from vehicle or vaccinated mice with diabetic splenocytes into NSG recipient mice. **(C**,**E)** Adoptive transfer of Tr1 depleted splenocytes isolated from vehicle or vaccinated NOD into NSG mice. **(A**–**C)** Data display the blood glucose levels for individual mice over the time. **(D**,**E)** Data presented as the Log-rank plot of the percentage of NSG mice that remained diabetes-free over the time after transfer. The differences between the group of mice vaccinated with combined therapy and vehicle was significant (**p < 0.01) by the log-rank (Mantel-Cox) test.

To evaluate the role of suppressive Tr1 cells in vaccine-mediated prevention of diabetes, adoptive transfer experiments (*in vivo* suppression assays) were performed with a focus on the Tr1 population. Transfer of pooled splenocytes from diabetic NOD mice resulted in that all NSG mice were developed diabetes within 50 days of transfer (Figure 6A). Co-transfer of 1 x 10^5^ CD4^+^CD49b^+^LAG3^+^ Tr1 cells from vaccine-treated animals with diabetic splenocytes (1 × 10^6^) resulted in a significant decrease in diabetes. Indeed 75% of recipient mice were protected for as long as 80 days post-transfer (Figure 6B). However, following co-transfer of the Tr1 cells from vehicle-treated animals, 75% of recipient mice become diabetic after 50 days of transfer (Figure 6B). Figure 6D shows significant prevention of diabetes in mice given Tr1-cells isolated from vaccinated mice compared with mice given the Tr1 isolated from vehicle-treated mice (Log-rank (Mantel-Cox) test, *p* = 0.003). To corroborate this finding, splenocytes from vehicle or vaccine-treated animals were depleted of Tr1 cells prior to transfer. Depletion of Tr1 cells from vaccine or vehicle-treated animal splenocytes resulted in similar effect as 3 out of 10 recipients becoming diabetic (Figure 6C). More specifically depletion of Tr1 splenocytes led to an increase in the incidence of diabetes (Figure 6E). Collectively, the results indicate that *Salmonella*-based combined therapy increases the frequency of functional Tr1 that suppress proliferation of responder T cells *in vitro* and disease specifically *in vivo*.

## Discussion

We and others reported that an antigen-specific combination therapy induced tolerance in a diabetic NOD mice model (38–40). Autoantigen-specific strategies aim to restore the loss of tolerance that underlies T1D with fewer adverse-effects than non-specific immunosuppression. Therefore, the combination of two approaches has been tested to provide specific and potent response (41). In our previous study, we developed an oral *Salmonella*-based combined therapy using PPI+TGFβ+IL10 combined with low doses of anti-CD3 mAbs that prevent T1D in NOD mice (38). This *Salmonella*-based combined therapy was safe and effective in inducing long-term normoglycemia in mice with new-onset diabetes.

Our goal in the present study was to evaluate the therapeutic effect of *Salmonella*-based combination therapy on new-onset of diabetes and to assess the role regulatory cells played in this process. Herein we showed that oral *Salmonella*-based combined therapy of PPI+TGFβ+IL10 with low dose of anti-CD3 prevented and effectively reversed new onset of T1D in NOD mice. This reversal effect on diabetic mice appeared to be specific and require the presence of autoantigen PPI, as no effect was found in animals exposed to the non-diabetic *Listeria* antigen (Llo) under the same vaccine-induced tolerogenic condition. This indicated that the reversal of diabetes via oral *Salmonella*-based combination therapy represents an antigen-specific response. Furthermore, the combined therapy increased the levels of the tolerogenic cytokines IL10 and TGFβ in the peripheral circulation of vaccinated mice, as well as inhibiting inflammatory cytokine levels after found in conditional medium of peptide-stimulated splenocytes.

Previously we observed a significant increase in the number of CD4^+^CD25^+^Foxp3^+^ Tregs in the spleens and PLNs of NOD mice vaccinated with combination therapy (38). In the present study, splenocytes isolated from vaccinated mice provided more protection from the development of diabetes when administered to NSG mice than cell obtained from vehicle-treated mice, indicating that the splenocytes contain a suppressor cell fraction that mediates the dampening of autoimmunity. Furthermore, we found that the vaccine-mediated therapeutic effects were accompanied by a robust increase in Tregs and an increase in Tr1 cells suggesting this may be, in part, the mechanism by which the vaccine functions. Furthermore, the vaccine-mediated induction of Treg and Tr1 was antigen-specific and essential to suppress autoimmunity. Tregs, formally known as suppressor T cells, especially naturally occurring CD25^+^CD4^+^ Tregs, in which expression of Foxp3 occurs in the thymus, maintain immunological self-tolerance and modulate immune homeostasis (42, 43). Depletion or deficiency in Tregs numbers or activity may cause or exacerbate diabetes in NOD mice (44).

Adoptive transfer of Treg splenocytes from vaccinated mice have suppressive activity more than the one from vehicle-treated mice. This suppressive activity of Tregs was only enough to prevent the development of diabetes in <40% of NSG mice. Adoptive transfer of Treg-depleted splenocytes from vaccinated NOD mice had only a partial impact on the development of diabetes in NSG mice, suggesting that the vaccine derived effect could be from other types of suppressor T cells. This hypothesis is supported by our finding that the partial effect mediated by Tregs in the vaccine-driven prevention of diabetes in NSG mice appears to be due to an associated increase in Tr1 cells. However, it is possible that complete prevention and reversal of diabetes in NOD mice by the *Salmonella* combinatorial therapy is secondary to induction of both Tregs and Tr1 cells.

Tr1 cells have been shown to induce tolerance in mice and humans by regulating cytokine secretion and proliferation of T effector cells (45). We found significant increases in the number of CD4^+^CD49b^+^LAG3^+^ Tr1 cells in spleens of NOD mice vaccinated with *Salmonella*-based combined therapy. Furthermore, *in vitro* stimulation of these Tr1 cells displayed insulin antigen-specific increases in cytokine production of IL10. Tr1 cells isolated from vaccinated NOD mice have strong *in vivo* suppressive activity after adoptive transferd into NSG mice. This suppressive activity of Tr1 was effective enough to prevent the development of diabetes in 75% of recipient NSG mice. In addition, Tr1-depleted splenocytes isolated from vaccinated mice prevented diabetes in 30% of NSG mice. In addition these Tr1 cells retained antigen-specific response if derived from mice receiving the complete combination therapy. Type 1 regulatory (Tr1) cells are in part, defined by their ability to secret significant amounts of cytokines such as IL10 and TGFβ (15, 25, 46). IL10 has anti-inflammatory action and is involved in maintaining immunological tolerance (47). It is reported that IL10 induces long-lasting tolerance and mediates differentiation of long-living antigen-specific Tr1 cells (15, 48). Such cells could then be attracted to the site of inflammation to suppress proliferation of T effector cells. The ability of IL10-producing Tr1 cells to restrain the activation of effector immune cells during autoimmune responses underscores their crucial role in maintaining immune tolerance (15). Our data proved that combination therapy with IL10 is required for long term Tr1 induction. These observations are supported by studies from others (23) where IL10 receptor signaling was found essential for Tr1 cell function *in vivo*. The same studies showed that IL10 maintains IL10 production in Tr1 cells via activation of p38 MAP kinase.

In conclusion, oral *Salmonella*-based vaccine is an attractive combined therapy to prevent T1D and to revert new onset diabetes in NOD mice. Our study showed that *Salmonella*-based combination without either TGFβ or IL10 was not effective. The therapeutic effect of combined therapy was mediated by the induction of both Treg and Tr1 cells which suppress the autoimmunity. Additionally, we found Tr1 cells more crucial than Treg cells in limiting and mitigating diabetes. These regulatory cells induced by combination therapy are autoantigen-specific and are essential to suppress the autoimmune responses specifically. This is the first demonstration of the role of Tr1 cells in the mechanism of *Salmonella*-based combined therapy to induce tolerance in NOD mice.

## Author Contributions

JM, KF, and MH conceived and designed the experiments. JM, PG, JR, NG, JC, and MH performed the experiments. JM, KF, and MH analyzed the data. JM, FK, KF, and MH wrote the paper.

### Conflict of Interest Statement

The authors declare that the research was conducted in the absence of any commercial or financial relationships that could be construed as a potential conflict of interest.
