# Supplementary material for: Reversal of New Onset Type 1 Diabetes by Oral Salmonella-Based Combination Therapy and Mediated by Regulatory T-Cells in NOD Mice
Source: Front Immunol. 2019 Feb 26;10:320. doi: 10.3389/fimmu.2019.00320 (PMC6400227; doi:10.3389/fimmu.2019.00320)
Supplement: Supplementary file 1 [file Presentation_1.pdf]

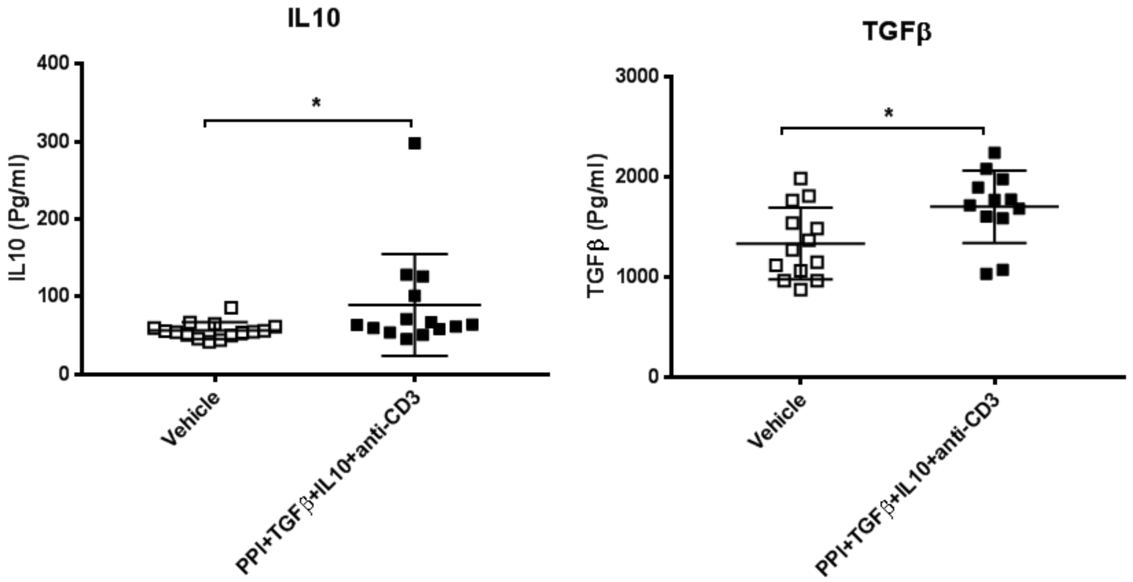

Supplementary Figure 1. *Salmonella*-based combined therapy increases tolerogenic regulatory cytokines. Serum was collected from vaccinated and vehicle treated NOD mice at 60 days post-vaccination and the levels of IL10 and TGFβ were measured using Mouse ELISA Kit. Data presented as means ± SD from 2 independent experiments. Significant differences were determined by Mann-Whitney *t* test.*P < 0.05 differences between combined therapy and vehicle treated.

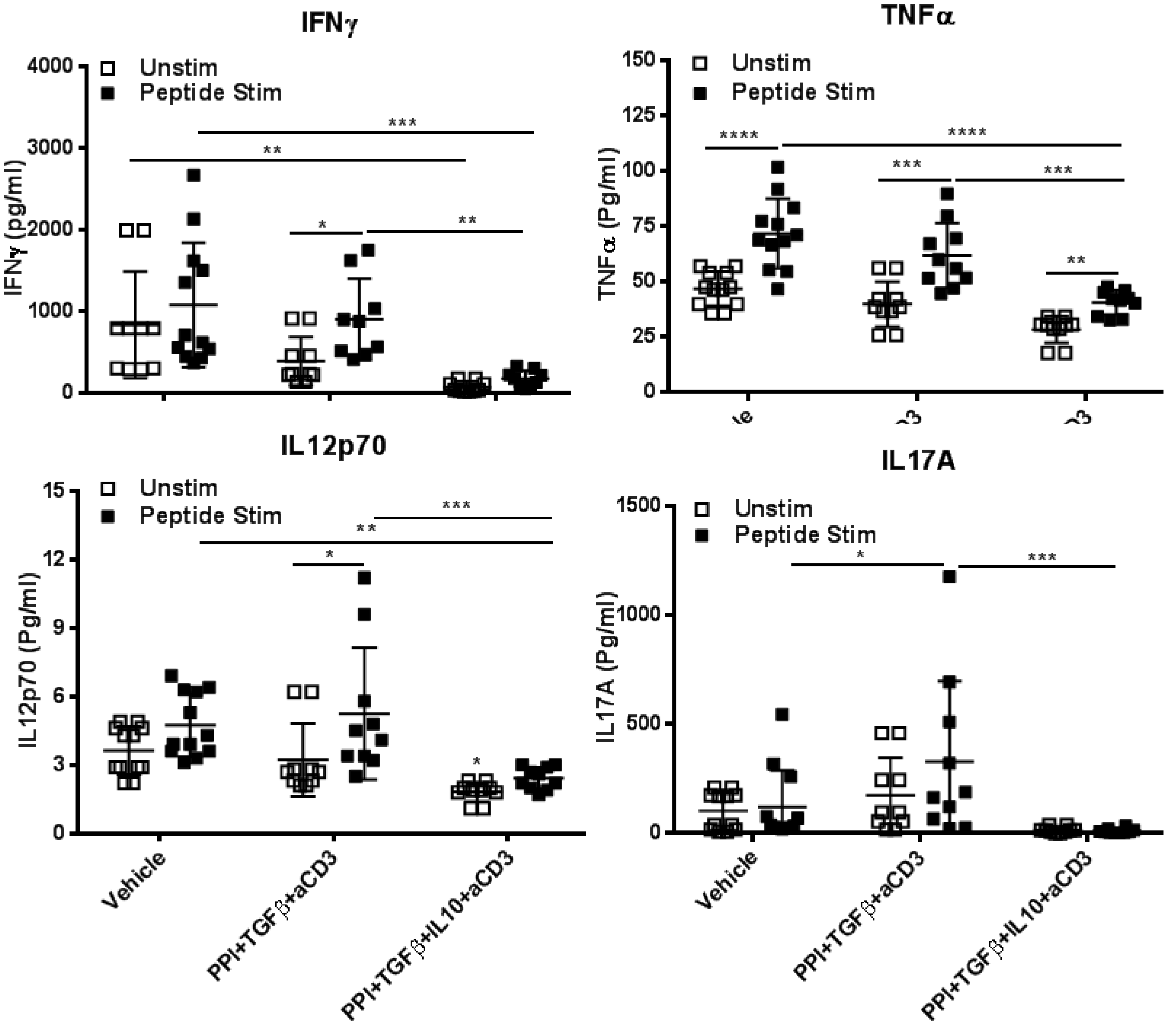

Supplementary Figure 2. Combined therapy specifically inhibite secretion of inflammatory cytokines. Splenocytes were harvested from NOD mice at day 60 post-vaccination and re-stimulated with insulin (B9-23) peptide *in vitro* for 72 h. Secretion of IFNγ, TNFα, IL12p70 and IL17A were quantified by multiplex Luminex analysis of culture supernatants obtained from peptide stimulated or un-stimulated splenocytes. Data presented as means ± SD and obtained from 2 independent experiments. Statistical analysis using two-way. ANOVA shows the significance between combined therapy and vehicle group (*$p<0.05$, **$p<0.01$ ***$P < 0.001$, ****$p<0.0001$)

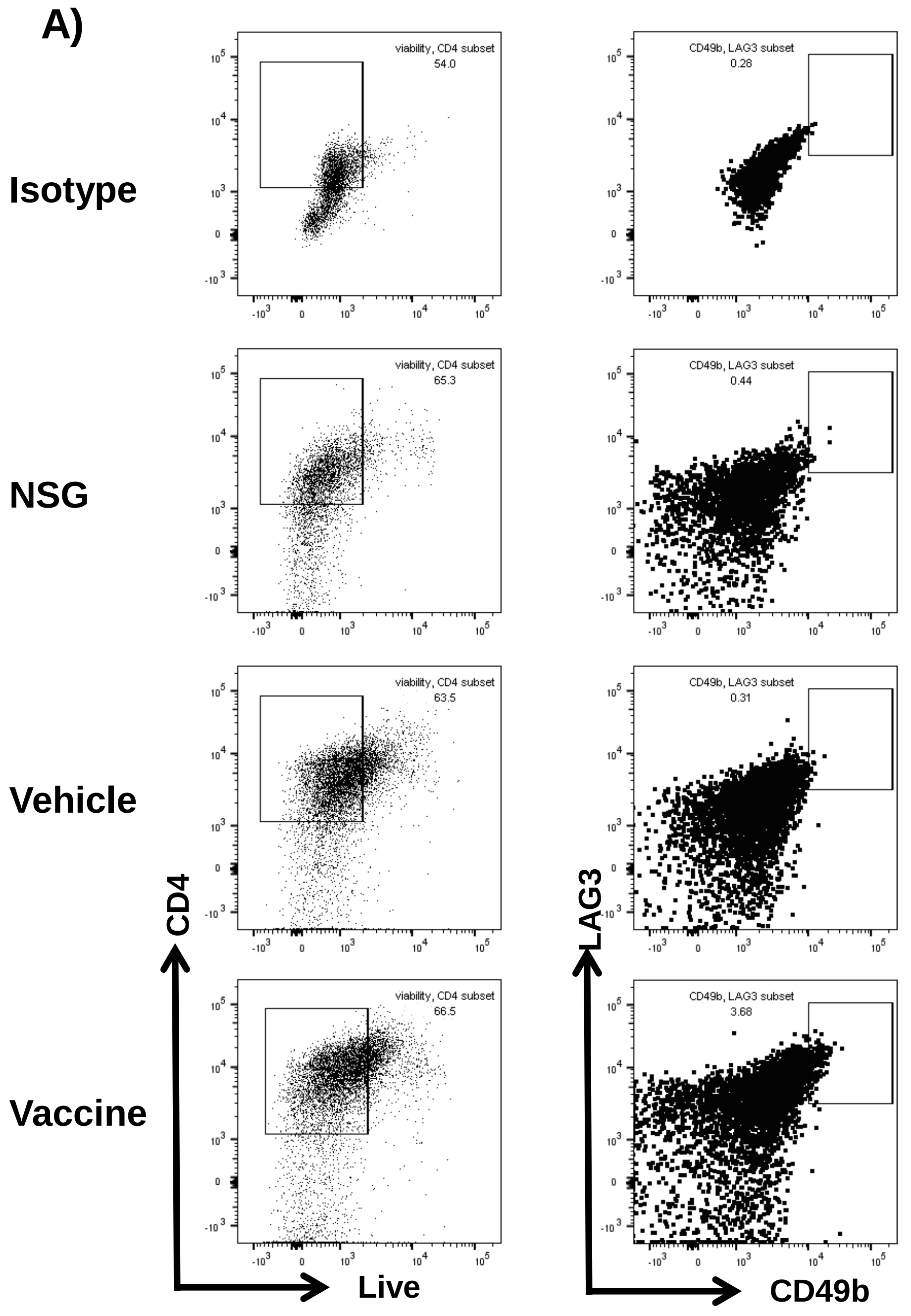

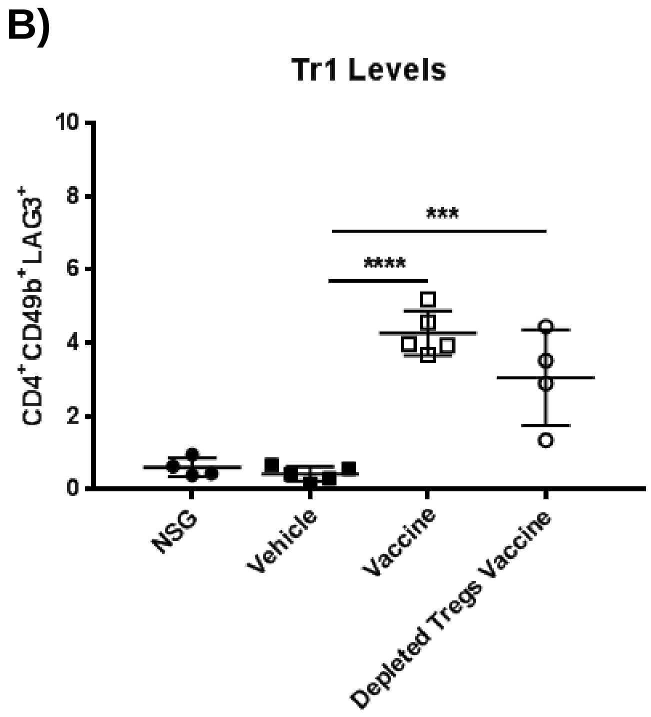

Supplementary Figure 3. Combined vaccine increases the frequency of Tr1 in NSG mice. Femal 7-8 weeks old of NSG recipient mice were adoptively transferred with indicated cell types. Splenocytes were isolated after 2 months post-transfer. Splenocytes from NSG mice used as negative control. **(A)** Representative FACS plots gated on live CD4 T cells indicate the frequency of $CD4^{+}CD49b^{+}LAG3^{+}$ T cells in the spleens of mice from the indicated groups. **(B)** Scatter plots of the percentages of $CD4^{+}CD49b^{+}LAG3^{+}$ Tr1. Data presented as means ± SD. Statistical analysis using one-way ANOVA shows the significance between splenocytes from vaccine or Treg depleted splenocytes compared to splenocytes from vehicle group (***, $p<0.001$; ****, $p<0.0001$).
